# Pasta Enriched with Carrot and Olive Leaf Flour Retains High Levels of Accessible Bioactives after In Vitro Digestion

**DOI:** 10.3390/foods12193540

**Published:** 2023-09-22

**Authors:** Veronica Conti, Chiara Piccini, Marco Romi, Patrizia Salusti, Giampiero Cai, Claudio Cantini

**Affiliations:** 1 Department of Biological, Geological and Environmental Sciences, University of Bologna, 40126 Bologna, Italy; 2 Department of Life Sciences, University of Siena, 53100 Siena, Italy; chiara.piccini1989@gmail.com (C.P.); marco.romi@unisi.it (M.R.); giampiero.cai@unisi.it (G.C.); 3 National Research Council of Italy, Institute for Bioeconomy (CNR-IBE), 58022 Follonica, Italy; patrizia.salusti@gmail.com (P.S.); claudio.cantini@ibe.cnr.it (C.C.)

**Keywords:** polyphenols, antioxidant, vitamin E, pasta, digestion

## Abstract

The aim of this research was to evaluate the levels of antioxidants and polyphenols in pasta enriched with either carrot or olive leaf flours after simulating gastrointestinal digestion. Pasta samples were prepared with fixed amounts of carrot and olive leaf flours (15% and 6% of the total mixture, respectively). We measured the antioxidant capacity and polyphenol content at different stages of the pasta production process, starting from the initial flour to the cooked pasta, and tested samples of the liquid component and solid waste resulting from the digestion process. The antioxidant activity was measured by the FRAP method, while the polyphenol content was measured by the Folin–Ciocalteu method. Vitamin E contents were measured by HPLC. The pasta enriched with carrot (1.26 ± 0.05 mmol/100 g) and olive leaf (2.9 ± 0.07 mmol/100 g) exhibited higher antioxidant power compared to the unenriched pasta (0.8 ± 0.1 mmol/100 g). The polyphenol content followed a similar trend, with values of 131.23 ± 3.08 for olive flour-enriched pasta, 79.15 ± 1.11 for carrot flour-enriched pasta, and 67.5 ± 1.39 for the wheat-only pasta. The pasta samples maintained their antioxidant and polyphenol levels even after undergoing the simulated digestion process. Significantly, the liquid component of the pasta with olive leaf flours had the highest levels of antioxidants and polyphenols during all stages of the digestion process. According to the results of this study, pasta enriched with carrot and olive leaf flours shows promising potential for improving nutritional and functional properties by increasing antioxidant and polyphenol content. The samples were also evaluated by a sensory panel, which showed that fortification modified the perception of some organoleptic attributes without affecting the overall taste of the pasta.

## 1. Introduction

Pasta is a popular food worldwide due to its affordability, nutritional value, versatility, ease of preparation, suitability for dry storage, and appealing sensory characteristics [1,2]. Pasta comes in a variety of shapes and sizes and is a good source of complex carbohydrates that provide energy. It is also low in fat and sodium and contains some vitamins and minerals and a moderate amount of protein [3]. To make pasta dough, durum wheat semolina (*Triticum turgidum* var. durum Desf.) is mixed with water (with a water content of approximately 28–32% *w*/*w*) and subjected to mechanical energy. The process results in a crumbly dough, which is then used to produce fresh pasta on an industrial and artisanal scale. The resulting pasta can then be dried [4]. Historically, durum wheat semolina has been the preferred ingredient for pasta production due to its distinctive yellow color and unusually high protein content (10.9–13.5%). This protein content enhances functionality and ensures appropriate dough structural properties [1,5]. The nutritional composition of pasta reflects its original material, serving primarily as a source of carbohydrate and protein.

Despite its nutritional importance, pasta has a low concentration of bioactive compounds. These are natural, non-nutritive molecules present in small amounts. They have specific physiological effects on the human body that go beyond basic nutritional functions [6]. Pasta contains minimal amounts of fiber, vitamins, essential amino acids, and minerals. However, due to its global popularity, ease of preparation, and widespread consumption, pasta is considered an effective carrier of bioactive components such as proteins, phytochemicals, minerals, and vitamins [2]. According to Bustos [7], up to 10–15% of non-traditional ingredients can be incorporated without significantly reducing the quality of the pasta. However, the quality may vary depending on the ingredients and the pasta processing technology used. Pasta research has long explored the partial or complete replacement of durum wheat semolina with alternative cereal or vegetable flours, as well as animal-derived materials. In today’s industry, the integration of highly functional ingredients to enhance the value of pasta is an area of research that is constantly evolving and can attract new consumer segments [1], especially those who are becoming increasingly health-conscious.

Fortified pasta is a type of pasta that contains more nutrients than traditional pasta made with refined wheat flour. It may be an appropriate choice for people who want to increase their intake of fiber and other bioactive compounds, as well as for people with celiac disease or gluten sensitivity. Nutrients can derive from legume flour, rice, corn, or chickpea flour for additional health benefits [1,8,9]. In general, by incorporating bioactive compounds into foods, new functional foods can be formulated and enriched with molecules that have potential health benefits for humans [10,11,12] by preventing chronic diseases and improving overall health; in addition, incorporating bioactives (such as bioactive lipids and peptides [13,14,15]) into traditional foods can provide a more accessible and convenient method of consuming beneficial molecules. Wheat breads have already been supplemented with extracts of broccoli [16], hawthorn, soybean, and onion peels [17], and even saffron [18], while pasta has been enriched with extracts from several sources [2] and even from mucilaginous seeds [19]. The market share of wheat products supplemented with functional molecules varies depending on the product category, regional factors, and the type of functional molecule. Nevertheless, the need for functional wheat-based products is likely to increase in the coming years as consumers increasingly focus on health and wellness.

Given the importance of wheat-based products in Italian cuisine and the Mediterranean diet [20], we enriched pasta with bioactive components that can confer functional properties. Improving food quality is also an important part of the Tuscany Region’s programs to improve local agricultural practices. Such practices could contribute to the promotion of human health and the prevention of lifestyle-related diseases. In this study, durum wheat semolina (Senatore Cappelli variety) was combined with dried carrot and olive leaf flour. As a result, three types of pasta were created: a traditional one made with durum wheat semolina and two “innovative” types made with different percentages of carrot and olive flour. Therefore, we enriched the pasta with bioactives, in particular polyphenols and antioxidants, which can prevent and improve some lifestyle-related pathologies [21]. Olive leaves were chosen, even though they are not normally consumed as food, because they represent an environmentally sustainable option. In addition, the incorporation of olive leaf flours is well suited to enhance the nutraceutical properties of pasta, as olive leaves are rich in secondary metabolites, especially polyphenols and flavonoids [22]. It is worth noting that olive trees are widely cultivated in Italy (as well as in the Mediterranean basin) and are considered one of the most important economic crops in the Mediterranean region. Since there is no economic benefit to discarding olive leaves, they could be used to increase the nutraceutical content of foods. Carrots were selected as the second item because they are also a popular food and contain high levels of secondary metabolites, including carotenoids, polyphenols, and flavonoids [23].

Two aspects of this research were innovative. First, the fortification of wheat flour was conducted by directly adding olive/carrot flours (i.e., dried and then ground materials) rather than by adding olive leaf and carrot extracts. This allows for a more economical and sustainable product. Secondly, the nutraceutical analyses included the evaluation of pasta digestion products using an in vitro digestion approach. In a laboratory setting, the in vitro digestion protocol is used to replicate the physiological process of digestion by mimicking the sequential steps. This method is valuable for investigating nutrient interactions, bioaccessibility, and digestibility of all food components, including nutrients. In addition, it has been widely used to evaluate the antioxidant activity of bioactive compounds after digestion [24,25]. In this study, the in vitro digestion protocol was used to compare the antioxidant, polyphenol, and vitamin content of raw, cooked, and digested pasta to determine how effectively bioactive molecules can be absorbed by the human body. The direct utilization of flours that are abundant in biomolecules could enhance molecular bioaccessibility post-digestion. Further, sustainability is prioritized using olive leaves, a waste product with varying quality characteristics. Since some ingredients added to pasta have been shown to reduce its cooking resistance and organoleptic quality, we also compared the sensory attributes of three types of pasta using a tasting panel to study the effect of fortification on perceived quality.

## 2. Materials and Methods

### 2.1. Production of Flour and Pasta

**Figure 1:**
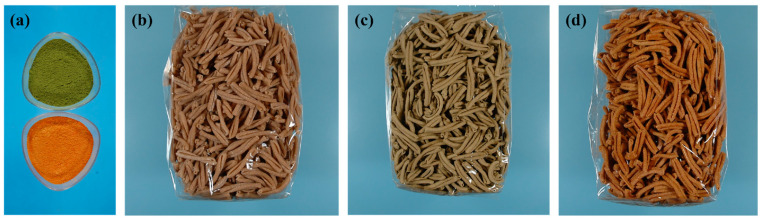
Flours and pastas used in this study. (**a**) Olive leaf flour (on the top) and carrot flour (on the bottom); (**b**) wheat-only pasta; (**c**) pasta supplemented with olive flour; (**d**) pasta supplemented with carrot flour.

The flour used for the traditional pasta was made from Senatore Cappelli durum wheat, stone-milled twice in a tumbler. The plant flours were prepared using a Biosec Pro dryer (Tauro Essiccatori, Camisano Vicentino, Italy). The carrot flour was prepared by drying commercially available carrots of PGI Carota dell’Altopiano del Fucino sticks (cv. “Maestro”, Vilmorin) in a dryer at 42 °C for the first two hours. The temperature was then reduced to 40 °C until the desired degree of drying was achieved (with a residual humidity of 6%). Similar steps were taken to obtain the olive leaf flour. Since the origin of the leaves could affect the chemical properties, we harvested one- and two-year-old leaves from cloned plants of the “Coratina” cultivar, grown under organic farming. After obtaining the dried plant materials, they were initially coarsely ground in an herbalist grinder mill (Albrigi Luigi, Stallavena, Italy) and then finely ground by an electric stone mill (Jumbo model, KoMo Gmbh & Co., Ltd., Hopfgarten, Austria). Three combinations of different percentages of flour were used to produce pasta. The first combination contained 100% Senatore Cappelli wheat, which was used to make the “traditional” pasta. The second combination was made with 85% Senatore Cappelli and 15% carrot flour and was used to make carrot pasta. The third mix was made with 94% Senatore Cappelli and 6% olive leaf flour and was used to make olive pasta. These percentages were chosen after a preliminary test designed to verify the rheological characteristics of the dough and the firmness of the finished pasta. All the preparations started with the mixing of durum wheat semolina with vegetable flour and water. The processes started in the upper tank for 25 min to hydrate the semolina, then moved to the lower tank for 30 min to create the gluten network of the dough (Iometti e Genghini, Roma, Italy, model IP60). At this point, the pasta came out of the die (Iometti e Genghini, Roma, Italy, model IP60) at a temperature of 26 °C. The pasta was then dried in a cabinet (A. Cozzi, S. Lorenzo di Parabiago, Italy, model EC24) until it reached the maximum residual humidity of 12.5%, as required by Legislative Decree no. 187 of 2001 (for the short “casarecce” pasta format, the drying time is 18 h at 40 °C). Figure 1 shows the olive and carrot flour (a), the wheat-only pasta (b), the olive pasta (c), and the carrot pasta (d).

### 2.2. In Vitro Digestion Protocol

To test whether the bioactives of the three pastas could be absorbed by the body, an in vitro digestion procedure simulating the physiological digestion of the pasta products was obtained from the literature [26,27,28]. This procedure resulted in six samples: three of the samples were digested fluid (the fluid theoretically absorbed by the body), and the other three were waste samples. For the in vitro digestion protocol, the dry pasta samples were boiled in 100 mL of unsalted water per 10 g of pasta, cooked for 10 min, drained, and collected for the next step. First, the pasta samples were placed in a mortar. Then, the mastication step, which is the initial phase of the digestion process, was simulated using a pestle. Next, gastric juice was mixed with pepsin (at a concentration of 2000–4000 U/mL; Merck, Darmstadt, Germany) at a ratio of 1:3 (*w*/*v*) and added to the finely ground sample; the mixture was incubated for 2 h at 37 °C in a water bath; the pH was adjusted to 3 with HCl 6 M. To simulate the intestinal phase, twice the volume of duodenal fluid was added to the previous mixture. Bile salts (at a concentration of 8 mg/mL; Merck, Darmstadt, Germany) and pancreatin (at a concentration of 200 U/mL; Merck, Darmstadt, Germany) were then added. The pH was adjusted to 7.0 with HCl 6 M. The new mixture was manually inverted and incubated for 3 h at 37 °C with stirring. After digestion, the samples were centrifuged at 1500 rpm (Eppendorf^®^ 5415D centrifuge, Hamburg, Germany) for 5 min. As a result of the centrifugation step, the solid phase, which is the digestion waste, was separated from the liquid phase, which contains the potentially assimilable nutrients.

### 2.3. Sample Extraction

All samples (obtained after digestion) analyzed in this study were stored at a temperature of −20 °C to preserve their organoleptic characteristics and prevent their deterioration. The dried pasta samples were ground with a mortar and pestle using liquid nitrogen until a fine powder was obtained for all three types of pasta. The cooked pasta samples were ground with a mortar and pestle. The pasta was prepared according to the recommended cooking methods. Flour was used directly without further preparation as it was already finely ground. The digested liquid and residual solids were used as they were obtained. The extraction method was performed by diluting 3 g of material (flour, raw pasta powder, cooked pasta, and residual solids from the digestion process) in 9 mL of 70% acetone (concentration ratio 1:3). For the digested liquid, 3 mL (equivalent to about 3 g) was added to 9 mL of 70% acetone. The mixture was then homogenized for 5 min using an IKA Ultra-Turrax^®^ T25 (Saint Louis, MO, USA) to combine the solution and further disaggregate the sample. The solution was sonicated for 20 min and then homogenized with Turrax for an additional minute to ensure complete disruption. The solution was then centrifuged at 4000 rpm (Eppendorf^®^ 5415D centrifuge, Hamburg, Germany) for 5 min to separate the pellet from the supernatant containing the nutraceutical compounds. To remove impurities, the supernatant was filtered through a 0.45 μm filter. Nutraceutical characterization was performed on all samples, and the preparation was repeated three times for each sample type to achieve statistical significance.

### 2.4. Measurement of the Antioxidant Power by the FRAP Method

The FRAP method [29] uses the reducing action of antioxidants on the TPTZ complex (2,4,6-tris(2-pyridyl)1,3,5-triazine) at an acidic pH to determine the antioxidant power. The binding of Fe²⁺ cations to the ligand causes the sample to exhibit an intense blue color. The absorbance measurement is directly proportional to the antioxidants present in the samples. Water was used in place of the samples for the control. An acetate buffer (2040 μL) was added to the tubes to achieve an acidic pH of 3.6. Then, 200 μL TPTZ, 200 μL ferric chloride (FeCl_3_), and 20 μL extracted samples were added. The samples were vortexed for several seconds and left at 37 °C for one hour to allow for a complete reaction. Samples were then read using a spectrophotometer (Shimadzu UV-1280) at 593 nm. The results were compared to a calibration curve previously constructed using ferrous sulfate solutions.

### 2.5. Phenolic Quantification by the Folin–Ciocalteu Method

The Folin-Ciocalteu (FC) method, as described by Ainsworth et al. (2007) [30], is based on electron transfer in an acidic environment. A complex of phosphotungstic and phosphomolybdic acids, which forms a blue chromophore, has a maximum absorption peak that depends on the phenolic composition. Water was used to replace the samples in the blanks. For each tube, 500 μL extract, 3950 μL distilled water, 250 μL Folin’s reagent (Sigma Chemical, St. Louis, MO, USA), and 750 μL sodium carbonate saturated solution (Na_2_CO_3_) were added. The samples were incubated at 37 °C for 30 min to complete the colorimetric reaction. After incubation, the samples were read on the spectrophotometer (Shimadzu UV-1280) at 765 nm, and the measurements were plotted on a calibration curve previously prepared with standard solutions of gallic acid (Sigma Chemical, St. Louis, MO, USA).

### 2.6. HPLC Analysis of Vitamin E

**Figure 2:**
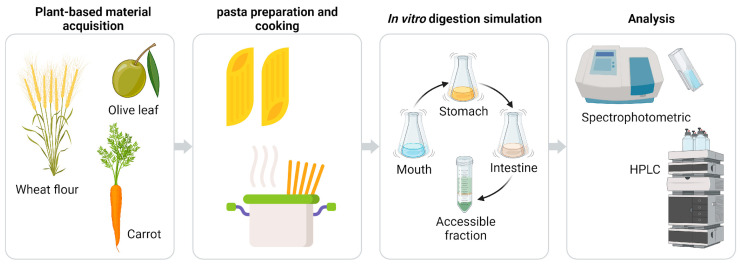
Overview of the main experimental steps for the analysis of flours from different sources. Wheat-based flours were mixed with either olive leaf flours or carrot flours. Prepared pasta underwent simulated in vitro digestion via oral, gastric, and intestinal methods. All samples, including flours, pasta, digestion waste, and accessible fractions, were subjected to spectrophotometric and HPLC analyses.

High-performance liquid chromatography (HPLC—Perkin Elmer Nelson 3200 Series) was used for quantitative and qualitative analysis of vitamin E. One gram of sample (a volume weight of 1 g was taken for the digested liquid) was diluted in 2 mL of pure ethanol, then the sample was homogenized with Turrax (IKA^®^-Werke GmbH & Co. KG, Staufen im Breisgau, Germany) for 5 min and finally centrifuged at 4000 rpm (Eppendorf^®^ 5415D centrifuge, Hamburg, Germany) for 10 min. Subsequently, 200 μL of the supernatant was collected and analyzed by HPLC. The chromatography setup consisted of a Waters 996 photodiode array detector and a 600E pump. A C18 250 × 4.6 mm (5 μm) column (SUPELCO Kromasil 100A-5u-C18 4.6 mm × 250 mm) was used for the analysis of vitamin E at a flow rate of 0.5 mL/min for 40 min. An injection volume of 50 μL was used, and the mobile (isocratic) phase consisted of 90% methanol, 10% acetonitrile, and 9 mM TEA (triethylamine). The concentration of vitamin E was then determined by standard curve calibration. All steps of the experimental procedure, such as plant source selection, pasta preparation and cooking, in vitro digestion, HPLC, and spectrophotometric analysis, are outlined in Figure 2.

### 2.7. Sensory Analysis

The dry pasta samples were boiled in 1000 mL of unsalted water per 100 g of pasta. The optimal cooking time was determined according to ISO 7304-2:2008 [31]. In our case, the cooking time was 10 min for the wheat-only pasta and the olive flour pasta, while 9 min was enough for the carrot flour pasta. The pasta was stirred gently every 2 to 3 min and allowed to boil. To stop the cooking process, 200 mL of cold water was added per liter of boiling water. After draining, the pasta was set aside to rest for 5 min. At this point, the portions were prepared. One hundred grams of Senatore Cappelli wheat pasta, carrot pasta, and olive pasta were randomly presented to ten sensory panelists for visual and tactile evaluation. The panelists were well-trained assessors belonging to the Chestnut Flour Italian Tasters Association. The group consisted of 5 women and 5 men, with an average age of 60 years. Before the pasta evaluation, the panelists were trained through three preliminary sessions to fully understand the evaluation method to be applied to the pasta samples and to evaluate anonymous samples of commercial pasta samples. During the olfactory-gustatory evaluations, the panelists were in a condition where they could not visualize the color of the pasta. Each panelist was asked to evaluate 30 g of each pasta type. Each sample was coded with 3-digit random numbers. To assess perceived intensity, panelists completed a sensory attribute form. Participants rated three visual attributes and two tactile attributes (firmness, elasticity, chewiness, adhesiveness, and stickiness) on a rating scale ranging from 1 (little) to 7 (very). Another part of the study evaluated taste and odor descriptors such as odor intensity, semolina, pasta, cut grass, hay, flower, sweet, salty, astringent, bitter, semolina flavor, pasta flavor, herbaceous flavor, pumpkin flavor, and flower/fruit flavor, which were rated on a scale from 1 (indicating a very weak odor or flavor) to 7 (indicating a very strong odor or flavor). To characterize the sensory properties of the olive and carrot pasta samples in comparison with the wheat pasta sample, the experiment was repeated twice. Finally, the median of the ten scores assigned to each attribute was calculated, and then the qualitative/quantitative results were presented by means of spider plots.

## 3. Results and Discussion

This paper discusses the use of traditional Senatore Cappelli wheat flour mixed with olive and carrot leaf flours. Olive leaves are often considered waste, while carrots are rich in antioxidants. The simple method allows pasta makers to label “dried carrots” or “dried olive leaves” without chemical extraction or purification, compared to more expensive methods. The use of a simple dryer also makes the production of fortified flour economically and environmentally sustainable at both artisanal and industrial levels [32]. Our study also analyzed digested pasta to assess the extent of absorbable substances [24]. We have used static models based on batch incubations with fixed volumes and compositions of digestion fluids, although they do not take into account the kinetic aspects of digestion.

### 3.1. Functional Flours Maintain High Antioxidant Levels in Pasta

**Figure 3:**
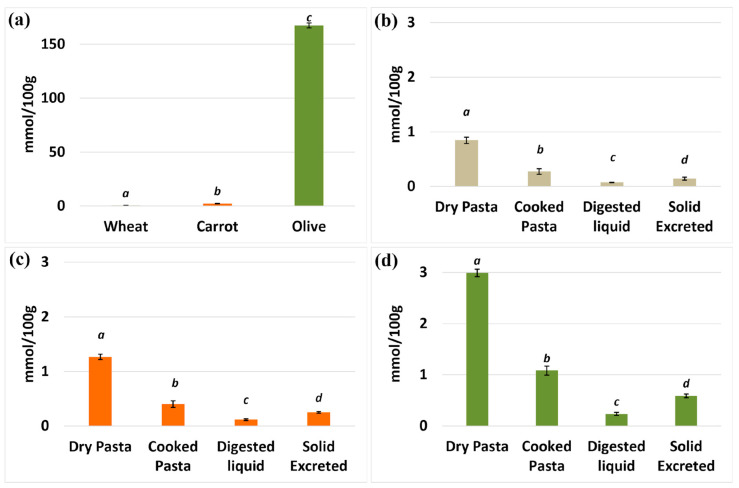
Antioxidant power in flour and flour-derived samples. (**a**) Antioxidant power (mmol/100 g) of basic flours: “Senatore Cappelli” durum wheat semolina (wheat), carrot, and olive flour. (**b**) The antioxidant power of dry pasta, cooked pasta, digested liquid, and solids excreted from “Senatore Cappelli” wheat flour. (**c**) Antioxidant power of samples from S.C. flour supplemented with carrot flour. (**d**) Antioxidant power of samples from S.C. flour mixed with olive leaf flour. Each different letter corresponds to a significant difference with a *p*-value ≤ 0.01. Note that the ordinate scale is different in panel (**a**).

First, we evaluated the antioxidant capacity of the three basic flours using the FRAP assay (Figure 3a). The data showed differences in the number of antioxidants among the flours. Olive and carrot flours had more significant antioxidant capacities than wheat. Among the two, olive flour had an exceptionally high antioxidant power (about 167.4 mmol/100 g), while carrot flour had a comparatively lower antioxidant power (2.3 mmol/100 g). Durum wheat flour “Senatore Cappelli”, on the other hand, had the lowest antioxidant power, only 0.46 mmol/100 g. This difference is maintained to a lesser extent when comparing the values obtained for dry pasta. For example, pasta made with “Senatore Cappelli” flour had an antioxidant capacity of about 0.8 ± 0.1 mmol/100 g (Figure 3b). In addition, pasta enriched with carrot flour had a significant (*p*-value ≤ 0.01) higher antioxidant capacity (1.26 ± 0.05 mmol/100 g) (Figure 3c). Moreover, dry pasta enriched with olive leaf flour retained its pronounced antioxidant capacity of about 2.9 ± 0.07 mmol/100 g (Figure 3d), significantly higher with respect to wheat-only pasta (*p*-value ≤ 0.01). It is suggested that the smaller increase in antioxidant capacity of pasta enriched with olive leaf flour is mainly due to the low percentage of such flours combined with Senatore Cappelli flour. When analyzing the antioxidant power of cooked pasta, we found that the values of antioxidant power were fairly consistent among the three types of pasta: cooked pasta made only with Senatore Cappelli flour had the lowest antioxidant power (0.27 ± 0.05 mmol/100 g), while the wheat–carrot mix had a slightly higher antioxidant power value. The pasta made with the wheat–olive leaf mixture showed the highest and most significant (*p*-value ≤ 0.01) value. The measurement of the antioxidant power in relation to the digested liquid and the secreted solid was of great interest to us because this analysis provided the value of the bioactives potentially accessible to the organism. The antioxidant power of the waste was consistently higher than that of the accessible digested liquid in all three cases, including Senatore Cappelli flour, the mixture with carrot flour, and the mixture with olive leaf flour (0.07 mmol/100 g in the liquid vs. 0.14 mmol/100 g in the waste for S.C.; 0.116 mmol/100 g in the liquid vs. 0.251 mmol/100 g in the waste for S.C./carrot mixture; 0.235 mmol/100 g in the liquid vs. 0.589 mmol/100 g in the waste for S.C./olive leaf mixture). This suggests that a significant portion of the antioxidants were not available and were lost. Nevertheless, the wheat/olive leaf mixture sample had more antioxidant power in the accessible and digested sample than the other two mixtures. Although the final antioxidant power in the digested liquid from the wheat/olive leaf mixture was significantly lower than in the equivalent dry pasta, it was still significantly higher (about two times) than in the accessible liquid from wheat flour alone. Even though pasta processing, cooking, and digestion may have reduced the antioxidant power in the accessible liquid, it was still maintained at a significant level in the accessible liquid from the wheat/olive leaf mixture.

### 3.2. Accessible Digestive Fluid Contains Higher Levels of Polyphenols

**Figure 4:**
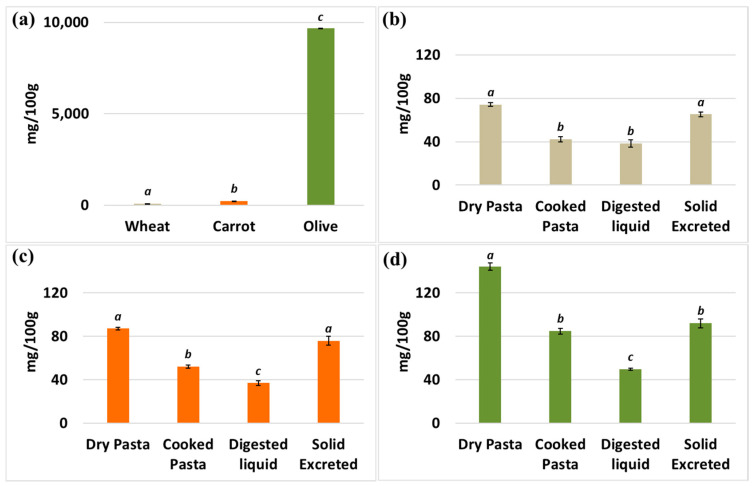
Content of polyphenols in flour and flour-derived samples. (**a**) Polyphenols (mg/100 g) of basic flours: “Senatore Cappelli” durum wheat semolina, carrot flour, and olive flour. (**b**) Polyphenols of dry pasta, cooked pasta, digested liquid, and solid excreted from “Senatore Cappelli” wheat flour. (**c**) Polyphenols of samples of S.C. flour supplemented with carrot flour. (**d**) Polyphenol content of samples of S.C. flour mixed with olive flours. Each different letter corresponds to a significant difference with a *p*-value ≤ 0.01. Note that the ordinate scale is different in panel (**a**).

Polyphenols (flavonoids, phenolic acids, lignans, stilbenes, and tannins) are characterized by one or more phenolic rings and exhibit antioxidant, anti-inflammatory, anticarcinogenic, and cardioprotective activities [33]. The profile of polyphenol content generally mirrors the profile of antioxidant levels (Figure 4). In particular, the olive leaf flour showed significantly higher polyphenol levels (around 10,000 mg/100 g) compared to the carrot flour and the Senatore Cappelli flour. After processing, the wheat dry pasta yield contained a polyphenol value just below 80 mg/100 g (Figure 4b), similar to that of the carrot dry pasta (Figure 4c). This suggests that the carrot made a very small contribution to the total polyphenol content. Dry pasta with added olive leaf flour had a significantly (*p*-value ≤ 0.01) higher polyphenol content with respect to other types of pasta, over 120 mg/100 g (Figure 4d), indicating that the addition of olive leaf flour, even at low levels, increased the polyphenol content. This is in contrast to the contribution of carrots to the polyphenol content of dry pasta. Even after the pasta was cooked, a similar ratio of polyphenol content was maintained among the different samples. Again, the cooked pasta enriched with olive leaf flour had almost double the polyphenol content of the other two samples (∼80 mg/100 g). However, these differences were less pronounced after the in vitro digestion test. In fact, in the case of Senatore Cappelli flour, the available liquid indicated a polyphenol value of about 40 mg/100 g. This value was almost the same as that of flour enriched with carrot flour and slightly but significantly (*p*-value ≤ 0.01) lower than that of flour enriched with olive leaf flour. These results suggest that after in vitro digestion of wheat/olive leaf pasta, only part of the polyphenol content is retained in the available liquid, in contrast to the antioxidant capacity. Polyphenols probably contribute less to the total antioxidant capacity. Similar to antioxidants, the ratio of polyphenol content between the accessible liquid and the secreted solid was almost consistently 1:2, indicating that a significant proportion of the polyphenols were inaccessible and therefore lost.

Previous studies have shown the potential to improve the nutritional and functional properties of pasta by fortifying it with various ingredients [34]. A study in which wheat flour was replaced with raspberry, boysenberry, redcurrant, and blackcurrant showed minimal changes in total polyphenols during processing [35]. In another study, sorghum and sorghum-enriched pasta showed a decrease in total polyphenol content after gastric digestion, while a slight increase was observed after duodenal digestion [36]. Fortification of semolina pasta with bioprocessed brewers’ spent grains resulted in higher protein digestibility and quality indices, as well as increased antioxidant activity [37]. Fortification of durum wheat pasta with encapsulated carrot flours resulted in an increase in carotenoid content [38], as well as improvements in antioxidant and anti-inflammatory activities [39]. The study conducted by Gull et al. (2018) [8] included carrot pomace (4%), which resulted in an increase in phenolic content and the antioxidant activity of functional pasta; however, it decreased after cooking. This finding suggests that a combination of millet flour and carrot pomace can be used to produce high-quality, nutrient-rich pasta. Locust bean flour has also been shown to increase the phenolic content and reduce the power of the pasta [40]. Salicornia europaea extract was added to fresh durum wheat pasta with no effect on the dough or cooking parameters [41]. However, it significantly increased the total phenolic and flavonoid content with increased antioxidant activity. The type of flour used, the way the pasta is prepared, and the source of the nutraceutical molecules may potentially influence published results. Although subject to variability, the majority of available data suggest that pasta fortification is a viable research direction. In vitro digestion seems to be a convenient method to quantify the accessible fraction of bioactives. The composition of the pasta may influence the polyphenolic content and the antioxidant efficacy of the resulting digestion fluid. Our research involved the use of a widely consumed wheat flour, Senatore Cappelli, for the production of pasta. We found that about two-thirds of the nutraceutical molecules were retained in the waste solids and therefore not accessible to the organism. Surprisingly, the addition of a minimal amount, only 6%, of olive leaf flour led to a significant increase in the polyphenol content and antioxidant power of the pasta, higher than the control group.

There is limited research on the fortification of pasta with olive leaf flours, and some studies have used other olive-derived by-products. Pasta has been enriched with olive pomace, resulting in a significant increase in total phenolic content and antioxidant capacity. While the cooking of the pasta affected the content of the total phenolic component, the cooked pasta supplemented with olive pomace still contained at least ten-fold higher content of polyphenols with respect to the control cooked pasta and about 5–6 fold higher antioxidant activity. Simultaneously, the enriched pasta requires less cooking time to reach optimum tenderness and has a lower cooking loss [42]. Another study investigated the effects of olive paste and olive mill waste water on bread and pasta, evaluating their sensory and chemical properties. The results showed that olive mill waste water slightly improved the chemical quality without affecting the sensory properties, while olive paste improved the chemical quality [34]. Olive leaf extract was used in the popular cereal-based food “taralli”, instead of white wine. The extract was then subjected to in vitro digestion to evaluate the antioxidant capacity and bioaccessibility of polyphenolic compounds. Results showed that replacing white wine with olive leaf extract increased polyphenol content and antioxidant capacity. However, oleuropein resisted digestion but was almost completely degraded in the intestinal phase [43]. These findings are in line with previous research indicating that incorporating a non-edible component of olive trees, such as leaves, can fortify and nutritionally enhance common foods such as pasta.

### 3.3. Decrease in Vitamin E Content during Pasta Preparation and Digestion

Most vitamins cannot be synthesized in the body, and dietary intake is essential. Vitamin E is a fat-soluble antioxidant that protects cells from oxidative damage and other diseases [44,45,46,47]. Natural (plant) sources of vitamin E provide alpha, beta, gamma, and delta tocopherol and tocotrienol from homogentisic acid [48]. Olive oil (especially extra virgin olive oil) is an example of a source of vitamin E [48]. In this study, given the high content of bioactives (polyphenols and antioxidants in general), we mainly analyzed olive flours, pastas, and digestive products for the content of vitamin E by HPLC analysis. Vitamin E was found at high levels in olive leaf flour (~10 μg/g) as well as in dry olive leaf pasta (~5 μg/g). After cooking, olive leaf pasta retained a significant level of vitamin E (~2.5 μg/g). Regrettably, after in vitro digestion, vitamin E was not detected in the digestion fluid of olive leaves or in the waste of olive leaves. Therefore, the majority of the samples were progressively deficient in vitamin E, which may be due to the manufacturing processes (pasta production and cooking processes) that caused vitamin degradation and a decrease in content. According to our data, vitamin E has been shown to be unstable during the processing and storage of extruded foods, primarily due to its susceptibility to oxidation [49]. Vitamin E has also been shown to decrease in content during pasta preparation, resulting in an almost 50% decrease [50]. In addition, it has been shown that vitamin E levels can vary after in vitro digestion depending on bile content and pasta composition [45].

### 3.4. Sensory Profile of Cooked Pasta

The taste, texture, and aroma of pasta can be affected by the incorporation of fortification ingredients, and few existing studies have focused on the sensory analysis of fortified pasta by investigating how functional ingredients affect the taste, texture, and nutritional aspects of pasta. The incorporation of red pepper and parsley leaf powders into pasta reduced cooking time and increased polyphenol content while maintaining overall acceptability [51]. The addition of broccoli leaf powder to durum wheat influenced technology and taste, but not on overall acceptability [52]. It is agreed that more research is needed to understand the sensory properties of fortified pasta [10,53].

**Figure 5:**
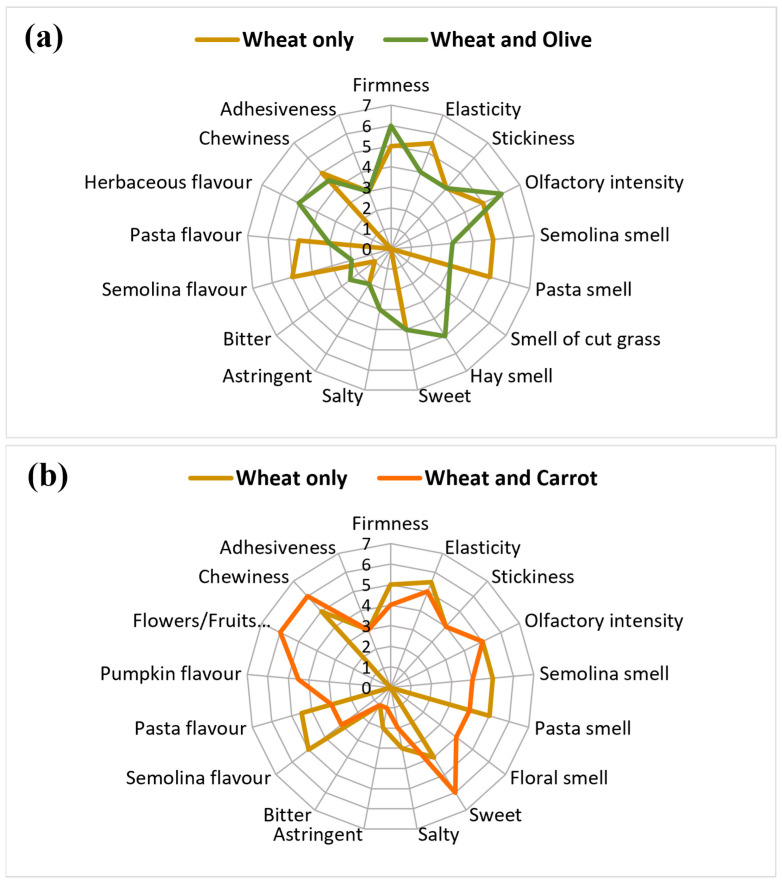
Radial graph of sensory attributes on a scale from 0 to 7 (where 0 means “nothing”, 1 means “little”, and 7 means “very”). (**a**) Radial graph of pasta enriched with olive flour (olive pasta-green) compared with pasta of Senatore Cappelli wheat (wheat pasta-light brown). (**b**) A radial graph of pasta enriched with carrot flour (carrot pasta—orange) compared with pasta of Senatore Cappelli wheat (wheat pasta—light brown). Each point on the graph represents the median of the scores assigned to each sample by ten panelists.

Compared to regular wheat pasta, the olive-enriched pasta had a more herbaceous flavor and less pasta and semolina flavor. In addition, the pasta with olives was perceived as having a strong hay and cut grass odor. This is not surprising, although it did not affect the overall taste of the olive pasta. In fact, the intensity of the smell, bitterness, and astringency were perceived as similar to those of wheat pasta. There were no differences in visual and tactile characteristics such as chewiness, adhesiveness, firmness, elasticity, and stickiness. The only difference was that the elasticity of the olive pasta was lower than that of the wheat pasta. This means that there was no effect on the cooking process of the pasta due to the addition of olive flour to wheat flour (Figure 5a). Based on the sensory analysis, carrot pasta and wheat pasta showed no significant differences. However, tactile and visual characteristics such as chewiness, adhesiveness, firmness, elasticity, and stickiness were similar to those of wheat pasta. Again, the use of carrot flour had no effect on the cooking process of the pasta. Compared to wheat pasta, carrot pasta, like olive pasta, showed some differences. For example, the flavors of flowers/fruits and pumpkin, as well as their floral aroma, were significantly greater than those of wheat pasta. Meanwhile, the pasta and semolina flavors were significantly lower than those of wheat pasta. However, such variations did not affect other parameters such as bitterness or astringency, as shown in Figure 5b.

## 4. Conclusions

The results of this study highlight several important conclusions. First, olive leaves can be used to fortify pasta while maintaining a high level of bioactives in the final product (either cooked pasta or the digestive fluid). This is an important achievement because olive leaves are usually waste that is not used as food or in food production. The use of olive leaves in pasta fortification represents a sustainable solution to reduce waste and maximize the nutritional value of the final product.

Secondly, this study provides strong evidence that the incorporation of carrot and olive leaf flours in pasta formulations improves the preservation of bioactive compounds during in vitro digestion. This suggests that the addition of carrot and olive leaf flours not only enhances the nutritional value of pasta but also contributes to its potential health benefits. However, olive leaf flour contributes more with respect to carrot flour for this purpose, maintaining a huge amount of healthy bioactive compounds. This opens up new opportunities for the development of functional food products that promote overall well-being and sustainability by effectively utilizing agricultural by-products. This finding suggests that fortified pasta can be effectively used in the diet to enhance the nutraceutical properties of pasta.

Thirdly, this study also highlights the use of valuable and cheap ingredients added to pasta, which increases the nutritional quality of pasta without having a major impact on the cost of the product. In fact, pasta is considered an economically convenient staple of the Mediterranean diet, and the purchase could be influenced by the final price, so fortification should take into account the additional cost to the standard pasta production chain.

Fourth, additional research is needed to explore the sensory aspects and applications of fortified pasta in the food industry, as well as the effects of long-term consumption on health. A deeper understanding of how different consumer groups perceive and prefer these fortified pasta products could provide valuable insights for product development and strategic marketing efforts. Not to forget, understanding the stability of these bioactives during storage is essential to maintaining their claimed health benefits.

## Data Availability

Data are contained within the article.
